# Supplementary figures and images for: Clinical Significance and Immune Infiltration Analyses of the Cuproptosis-Related Human Copper Proteome in Gastric Cancer
Source: Biomolecules. 2022 Oct 12;12(10):1459. doi: 10.3390/biom12101459 (PMC9599751; doi:10.3390/biom12101459)

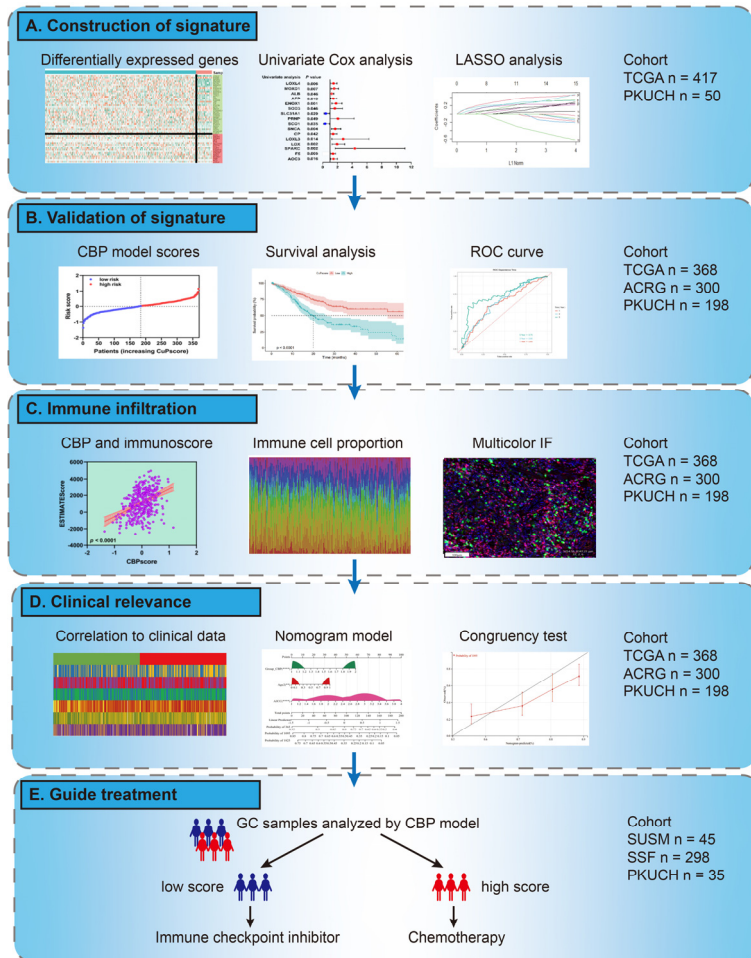

**Figure S1.** The workflow of this study.

Supplement: Supplementary file 1 [file biomolecules-12-01459-s001.zip › biomolecules-1932938-supplementary.pdf]
